# Progress in Disease of Digestive Organs

**Published:** 1900-12-08

**Authors:** 


### Progress in Disease of Digestive Organs.

**Ulcer.**—In a discussion at Ipswich, J. F. Payne[11] considered that ulceration of the stomach, like other digestive diseases, is becoming more common. He thinks pain is found in only a minority of the cases, vomiting in most of them, while hæmatemesis appears most often in non-fatal ones, since perforating ulcers rarely bleed. The distinction between acute and chronic forms, and the question whether water should be allowed by the mouth during rectal feeding were discussed. Cases of hæmatemesis were brought forward in which no ulcers existed, and the difficulties of diagnosis were acknowledged on all sides. Relief from pain is sometimes given by five grains of resorcin three times a day. For hæmorrhage in chronic ulcer Fenwick[12] advises rectal feeding for ten days or more, but in acute ulcers after 48 hours a little peptonised milk every two hours together with enemata for three days more. For severe hæmorrhage an icebag, turpentine, gallic acid, opium, kino, calcium chloride, hypodermic injection of 200 grammes of a 2 per cent. sterilised solution of gelatine in saline solution, or direct pressure by a tourniquet, have been recommended. E. Quintard,[13] referring to the various types of erosions and ulcers of the stomach, classifies them clinically as (*a*) ill-defined forms seen in uræmia, cirrhosis and fatal tubercular disease; (*b*) Diculafoy's microscopic ulcerations with no clinical symptoms till violent hæmorrhage sets in; (*c*) the classical round ulcer; (*d*) erosions where pieces of the mucosa are found in the stomach washings and the erosions are superficial only; (*e*) ulcers from chemical poisons; (*f*) those following burns; (*g*) the rare ulcers of syphilis, tuberculosis, &c. The erosions of class (*d*) arise, he thinks, in various pathological states, but usually show different symptoms to those of round ulcers.

For the analyses of stomach contents, F. Shufflebotham[14] advises (*a*) first to test for hydrochlor-proteids or free acids with Congo red or benzo-purpurin paper. (*b*) He then calculates the total acidity with soda and phenol-phthalein; next (*c*) the organic acids are removed from a portion of the contents by shaking up with an excess of ether for twenty minutes and allowing it to stand for some hours. The decanted ether carries off practically the whole of the lactic acid, which can be estimated by the same test. The residue contains only volatile acids, which are removed by boiling, and free HCl and hydrochlor-proteids (*d*) which are then calculated as before. The absence of lactic acid as shown by Uffelman's test will sometimes enable us to shorten the process. Maben,[15] indeed, thinks this test is unreliable, and prefers to use the ruby reaction given by sulpho-carbolate of zinc 4 grains, ferric chloride 2 grains, and half an ounce of water. If HCl and its compounds amount to about ·22 grammes per 100cc., and there is no lactic acid, then there are no serious lesions present, but merely nervous dyspepsia, or intestinal troubles. If the HCl is deficient with no lactic acid there may be chronic gastritis. If lactic acid appears in such a case there is deficient motility from cancer or dilatation. An excess of HCl is often seen in ulcer, and a deficiency in anæmia, Bright's disease, and, according to the writer, in cardiac trouble. It should be added that he finds the absence of *free* HCl is the rule even in health when digestion has made some progress.

**Gastroptosis** rarely occurs in males, it is the most frequent displacement of any abdominal organ, and often accompanies a floating kidney, and can best be diagnosed by the diaphane or inflation of the stomach. Achilles Rose,[16] in a very complete account of this condition, mentions its frequency in lean, slender persons with a long thorax (*e.g.*, some phthisical cases), in kyphosis, in persons with deficient motility of the stomach, or with certain herniæ or tumours, after the puerperal state, or after the cure of obesity. Again, relaxed suspensory ligaments of the intestines, and an abnormally moveable tenth rib have been said to produce it. As to treatment, Rose finds a broad rubber plaster most useful to support the abdominal walls. Various means of strengthening them have been tried, and massage with a heavy round ball seems as satisfactory as any. A neurasthenic condition is apt to exist in these patients, and any form of dyspepsia is exaggerated. Rose is strongly opposed to surgical treatment of loose kidneys. It should be noticed that the appearance of a pulsating aorta is sometimes due to gastroptosis. G. G. Campbell[17] describes a case of enteroptosis combined with factitious urticaria setting in after parturition three years before. Any stimulation of the skin caused red blotches and formication. The kidneys were prolapsed, the upper part of the abdomen was contracted and the lower distended.

**Dilatation.**—Acute cases are occasionally seen and may be obstructive or atonic. Bettman[18] thinks they are not infrequent, and quotes two instances where some obstruction in the duodenum after operation resulted acute dilatation, vomiting, and death. Nervous shoc from injury, overeating, or during convalescence from typhoid or other severe illness may cause more or less acute attacks with copious vomiting of retained and fermenting matter. Pyloric stenosis in not rare instances may, says Einhorn, be due to syphilis, and disappear under suitable treatment. Indeed, gastric ulcer may be produced in the same way, and such a possible origin should not be forgotten.

**Hyperchlorydria.**—Max Einhorn[19] thinks that this is the condition in quite half the cases of gastric dyspepsia. Discomfort an hour after meals with sometimes water brash and vague symptoms such as angina may be found, but a more reliable sign for diagnosis is the relief given by the ingestion of food. He does not believe in a strict diet, and merely reduces the amount of starch food, especially potatoes, seasoning, stimulants, and tea. He orders the meals to be small and frequent. Fats and sugar need not be cut off. Alkalies are given by him two hours after meals, *e.g.*, a drachm or more of soda; while bromides, electricity internally, and sprays of nitrate of silver are also useful. Mingot[20] says that atropine given thirty minutes before food at once reduces the excessive secretion of HCl, but these effects cease after a few days' use, and with increased doses other evils arise, so that on the whole he has ceased to employ this remedy. In hyperchlorydria and in other gastric conditions Einhorn has occasionally found mould fungi. Their identity was clear on microscopic examination, and they grew easily in the stomach washings. It is not so clear whether they cause much irritation, but they can be got rid of by lavage when the patient is fasting.

**Ulcer of the Duodenum.**—R. F. Weir[21] gives a most interesting account of this ulcer with a *résumé* of the literature of the subject. The ulcers are nearly always situated in the first two inches of the duodenum, very rarely below the bile duct with its alkaline discharge. They are generally single and on the anterior wall, 80 per cent. occur in males and are present in from 0·2 to 0·4 per cent. of all autopsies. Not infrequently they are met with after severe burns, and have been ascribed to septic infarcts or emboli; and, indeed, Lockwood shows that in 138 cases of burns treated antiseptically only one ulcer appeared. Hunter suggested that burns caused a change in the bile producing toluylendiamin, and that this led to the ulceration, but on the other hand they are usually found above the bile duct. In a few cases they are due to cancer. Symptoms before perforation are few and often unnoticed. Pain may occur in the right hypochondrium or to the right of the parasternal line two to four hours after food, or tenderness may be felt to the right of the twelfth thoracic vertebra, occasional vomiting or jaundice, and profuse melena with dark or bright red blood may all be seen. Thus a little pain, and perhaps some blood from the bowel, may be the only warning of illness before a fatal termination. In a few cases blood is vomited, leading to the supposition of a gastric ulcer or cirrhosis of the liver.

**Intestines.**—W. Reed[22] has reinvestigated the mode of absorption from the intestines, and demonstrates that it is due to the direct action of the epithelium, and not to mere filtration osmosis, or imbibition. Thus salts are taken up quicker than organic solids, and these faster than water, though in the last case osmosis may play some part. When the epithelium is destroyed, the process is lessened or stopped altogether. F. J. Waldo[23] reviews the causes and possibility of prevention of *summer diarrhœa*, which he regards as an acute infection. The bacteria which are the exciting cause are numerous, or at least unknown, and he inclines to think they multiply in the horsedung of the streets. It is peculiarly fatal to hand-fed infants, and the disease is conveyed very largely from patient to patient by contaminated clothing, hands, and furniture soiled by the excreta. The use of opiates is bad, calomel, castor oil, rectal injections of saline fluid, tannigen, and subgallate of bismuth are valuable remedies. The child may be kept for twelve hours on plain boiled water. He is anxious for the prevention of dust, the flushing of streets and courts, the storage of food in dust-proof safes, and the protection especially of milk from contamination. Shoemaker[24] urges the avoidance of mere opiates and astringents, and gives salicylate of bismuth with ipecacuanha, betanaphthol, salicyn, and irrigation. Others have obtained very good results with the liquor hydrargyri ♏x–xx in distilled water, and various forms of naphthol.

[11] Lancet, Aug. 11. [12] Ulcer of the Stomach and Duodenum, 1900. [13] Med. Rec., Sept. 15. [14] Lancet, Sept. 15. [15] Lancet, Sept. 29. [16] Post Grad., March. [17] Med. Rec., July 14. [18] Med. Chron., May. [19] Med. Rec., June 2. [20] New York Med. Jour., July 7. [21] Med. Rec., May 5. [22] Brit. Med. Jour., June 30. [23] Lancet, May 12—26. [24] Med. Bull., June.

